# Profiles of cannabis users and impact on cannabis cessation

**DOI:** 10.1371/journal.pone.0305088

**Published:** 2024-06-11

**Authors:** Amy L. MacQuarrie, Caroline Brunelle

**Affiliations:** Department of Psychology, University of New Brunswick Saint John, Saint John, New Brunswick, Canada; University of Connecticut Health Center: UConn Health, UNITED STATES

## Abstract

Although cannabis was legalized in Canada in 2018 and is one of the most used substances in Canada, few studies have examined how individuals with different patterns of cannabis use differ in their attempts to decrease or abstain from cannabis. The current study examined how groups of cannabis users, which were formed on the basis of demographic characteristics, substance use patterns, mental health symptoms, and self-reported quality of life differed on their experiences with cannabis cessation. A sample of 147 Canadian adult participants who had attempted to decrease or quit cannabis were recruited from the community (*n* = 84, 57.14%) and crowdsourcing (*n =* 63, 42.86%). Four profiles of cannabis users emerged using a Latent Profile Analysis: low-risk (*n* = 62, 42.18%), rapidly escalating high-risk (*n* = 40, 27.21%), long-term high severity (*n* = 35, 23.81%), and long-term lower severity (*n* = 10, 6.80%). Individuals in the rapidly escalating profile had attempted to decrease their cannabis use more times compared to other profiles. More participants in the long-term high severity group found their use stayed the same or got worse after their last cessation attempt, compared to the low-risk group where more individuals indicated their use stopped. The results of the current study indicate that cannabis users differ in their attempts at reducing or ceasing cannabis use and that they may benefit from different intensity of cannabis interventions.

## Introduction

The production, possession, and sale of cannabis products was legalized in Canada in 2018 under the Cannabis Act [1]. With the legalization of cannabis, researchers have called for increased empirical research on the etiology, course, and treatment of cannabis use disorder [2]. Cannabis use also has increased because of the COVID-19 pandemic, both in Canada [3, 4] and elsewhere [5, 6], with a scoping review indicating that coping with loneliness and symptoms of mood and anxiety disorders accounted for increased use [7]. Cannabis is the second most commonly used substance by Canadians after alcohol, and it is estimated that approximately 1 in every 11 cannabis users will progress to developing a cannabis use disorder (CUD) [8].

Unsuccessful attempts at reducing or controlling use is a symptom of CUD according to the DSM-5 [9]. Across the overall adult US population, the prevalence of this symptom was 3.3% in 2017 [10]. Although there is no known data on how many cannabis users desire to reduce or become abstinent from cannabis, it is unlikely that the desire to cut down cannabis use is limited to individuals with CUD. Non treatment seeking cannabis users report making on average 4 quit attempts over their lifetime [11]. However, individuals often transition from reduction and abstinence goals interchangeably and users with reduction and abstinence goals are similar in terms of cannabis outcomes [12]. For this reason, it is worthy to examine cannabis users with either reduction or abstinence goals within the same study.

Cannabis users who have attempted to reduce or stop cannabis use are a rarely examined group in research. In one of the rare studies examining daily cannabis users who wished to reduce or cease cannabis use, Hughes and colleagues [13] found that attempts at reducing cannabis were more frequent than attempts at abstinence, that many users (92%) failed at abstinence attempts and that few participants had accessed formal treatment to assist them. In a three-year longitudinal study of individuals with CUD [14], it was found that daily cannabis use at onset as well as use of other substances were associated with greater odds of full or partial (cut down use) remission from cannabis at study completion. Furthermore, a retrospective study found that older age, being female, having engaged in psychological treatment and not experiencing an increase in cannabis use in the first three years of use were predictors of cannabis cessation [15]. Although these studies are informative as they suggest some individual variables associated with cannabis outcomes, no study has explored how specific types of cannabis users may experience cannabis reduction or cessation differently.

Few (10%) cannabis users enter formal treatment and psychotherapeutic cannabis interventions have moderate efficacy at reducing cannabis use and modest efficacy at reaching complete abstinence [16]. One potential avenue to improving treatment efficacy is further consideration of individual differences of personal, biological and social factors in implementing treatment targets [17]. Treatment matching has been suggested as one avenue to improve cannabis treatment outcomes [18]. Typologies of substance users, which separate substance users into groups that share similarities, may offer guidance in matching treatment-seeking individuals who use substances to the most appropriate levels of intervention [19]. The objectives of this cross-sectional study are therefore twofold. The first aim is to group cannabis users who reported having ever attempted to reduce or cut down on their use of cannabis based on characteristics such as their patterns of substance use, their mental health symptoms and their quality of life. The second objective is to subsequently examine their experiences with cannabis reduction and cessation, to determine if they specifically differed in the number of quit or cessation attempts and their access to formal treatment interventions. It was hypothesized that three groups would emerge: A group consisting of individuals who use cannabis at a low frequency, a moderate use group in terms of frequency and severity of use and a group experiencing high use and mental health comorbidity impacting quality of life. As no previous studies have compared cannabis user profiles on cannabis cessation, no formal hypothesis has been formulated on such comparisons.

## Method

The study was advertised on social media platforms, including sites targeting cannabis users (e.g., Twitter, Reddit, Facebook, Weedlife) as well as on a crowdsourcing site (Amazon Mechanical Turk [mTurk]), which has previously been reliably used to recruit substance-using populations [20, 21]. In order to participate, cannabis users had to report at least one cannabis reduction or cessation attempt but this attempt did not need to have been successful. Participants accessed the informed consent form and survey by clicking on a link to the survey, which was hosted on Qualtrics, an online survey platform. After completion of the survey, community participants were invited to enter their contact information on a separate survey in order to enter a draw to win one of several gift cards and MTurk participants were renumerated with $2US. The study was approved by the institution’s research ethics board.

### Measures

#### Demographics

Demographic information was collected using a self-developed questionnaire that assessed participants’ age, ethnicity, gender identity, occupation, education, relationship status, and total personal income.

#### Substance use patterns

Substance use variables were assessed using three self-reported measures: the Addiction Severity Index (ASI) [22], a modified version of the Daily Sessions, Frequency, Age of Onset, and Quantity of Cannabis Use Inventory (DFAQ-CU) [23], and a checklist based on the DSM-5 Cannabis Use Disorder criteria (DSM-CUD) [9].

The ASI [22] asks participants to rate their frequency of use of substances from never, past year, past 30-day to daily/almost daily use. In this study, participants were asked to select substances that they had ever used and indicate if they were prescribed by a physician. Substances included alcohol, nicotine (e.g., cigarettes, e-cigarettes), opioids (e.g., heroin, “Oxy”, Dilaudid, Fentanyl, T3/4s), cocaine (e.g., freebase, powder, “crack”), amphetamines (e.g., crystal meth, “speed”, “ice”, Adderall), cannabis (e.g., plant, oil, shatter, edibles, hash), hallucinogens (e.g., ecstasy, MDMA, PCP, LSD), and inhalants/solvents (e.g., nitrous oxide, gas, glue, paint thinner). The total number of substances ever used was tallied to reflect polysubstance use. Furthermore, a count of the total number of ‘legal’ (alcohol, nicotine, cannabis) and ’illicit’ (opioids, cocaine, amphetamines, hallucinogens, sedatives, and inhalants/solvents) substances ever used was computed.

The DFAQ-CU Inventory [23] is designed to assess cannabis usage, including the age of onset, frequency of use, current and past cannabis use patterns, and method of use. The measure has shown good to excellent reliability and excellent convergent validity with other cannabis instruments [23]. For this study, specific items of the DFAQ-CU were used to operationalize age of onset and frequency of use. For age of onset, the question “How old were you when you FIRST tried cannabis” was used. For frequency, three items were selected, “How many years in total have you used cannabis”, “Approximately how many days of the past month did you use cannabis”, and “Which of the following best captures the average frequency you currently use cannabis?” Categories for average frequency were collapsed for ease of interpretation: I do not use cannabis, less than once a year, yearly (i.e., once a year, 2–4 times per year, 6 times per year, 12 times per year), monthly (i.e., 2–3 times a month), weekly (i.e., once a week, twice a week, 3–4 times a week, 5–6 times a week), daily (i.e., once a day), and more than once a day.

The DSM-5-CUD [9] consists of a 12-item dichotomous (yes/no) checklist designed to assess past week DSM-5 symptoms of CUD. Scores range from 0–11. Similar to the DSM-5 [9], scores of 2–3 symptoms would be considered a mild CUD, a score of 4–5 indicates a moderate CUD, and a score of 6 or above is indicative of the presence of a severe CUD. The internal consistency of past week DSM-5 CUD symptoms (α = .87) was good. This SUD assessment method has been used in previous research [24, 25]. Self-reported assessment of SUDs has also been found to be more accurate than interview format [26].

#### Quality of life and mental health symptoms

Participants’ quality of life and overall mental health was assessed using The Quality-of-Life Enjoyment and Satisfaction Questionnaire—Short Form (Q-LES-Q-SF) [27] and the DSM-5 Self-Rated Level 1 Cross-Cutting Symptom Measure—Adult (DSM CC) [9].

The Q-LES-Q-SF is a 16-item questionnaire designed to assess various aspects of an individual’s quality of life within the past week (physical and mental health, mood, relationships, ability to function in daily life, work, etc.) [27]. Previous studies have indicated that it is a reliable and valid measure of quality of life [28–30]. Items related to the ability to get around physically, the impact of vision, and use of medication were excluded from the measure, as they were irrelevant to the context of the study, resulting in 13 items. The Q-LES-Q-SF is scored by summing items to generate a raw score and then using a formula to transform it into a percentage maximum score [27]. Total scores could range from 12 (one item assessing overall life satisfaction is excluded from the total score) to 60. Higher scores are indicative of improved quality of life and life satisfaction. Although three items were omitted, the internal consistency of this instrument was excellent (α = .92).

The DSM-5 CC Measure [9] is a self-reported 23-item measure of mental health symptoms across various domains (i.e., depression, anger, mania, anxiety, somatic symptoms, suicidal ideation, psychosis, sleep problems, memory, repetitive thoughts and behaviours, dissociation, personality functioning, and substance use) in the past two weeks. It captures symptoms that are not tied to one specific DSM disorder but rather are meant to cut across diagnostic categories. As such, it is not meant as a diagnostic tool but rather as a tool to inform clinical decision making regarding mental health symptomatology. A body of literature [31, 32] has established that mental disorders are better described along two broad dimensions rather than as single diagnostic entities. Specifically, most disorders fall along an internalizing (e.g., depression, anxiety disorders) and externalizing dimension (e.g., psychopathy, aggression, substance use disorders, ADHD). Additional research [33] suggests the existence of a third dimension inclusive of thought disorders (e.g., psychotic disorders, bipolar 1 disorder). For these reasons, in the current study, the DSM-5 CC anger and substance use subscales were combined to examine “externalizing” behaviours in participants and an “internalizing” symptoms variable was created by merging the depression, anxiety, and suicidal ideation subscales. Finally, the DSM-5 CC mania and psychosis subscales were also merged. Scores on each item range from 0 (none, not at all) to 4 (severe, nearly every day). In the current study, the internal consistency of the instrument was excellent (α = .93).

## Statistical plan

Prior to data cleaning, a total of *N =* 253 cases were available. Cases with more than 30.00% total missing data were removed from the data set, as well as outliers that exceeded +/- 3 standard deviations or were discontinuous with the rest of the data. Individuals who had never used cannabis, were younger than 19 years or had never attempted to decrease or quit cannabis were also excluded from participating in the survey. A total of *N =* 147 participants were retained in the dataset (ccommunity participants, *n* = 84, 57.14%; crowdsourcing participants, *n* = 63, 42.86%). Analyses were completed using the Statistical Package for the Social Sciences software, version 28 [34] and R [35]. Demographic variables, age of onset of cannabis use and frequency of use variables from the DFAQ-CU, current DSM-5 CUD symptoms, DSM-5 CC internalizing, externalizing and psychosis/mania scores, as well as the Q-LES-Q-SF percent maximum score were included in a latent profile analysis. Follow-up analyses using chi-squares, ANOVAs, and ANCOVA were conducted to examine group differences on latent profile variables as well as cannabis reduction and cessation variables.

## Results

### Sample characteristics

The sample was composed of *N* = 147 Canadian adults (over the age of 19 years) who self-identified as having previously attempted to decrease or quit cannabis use at least once. Participants mostly identified as men (*n* = 103, 70.07%), of European origin (*n* = 98, 66.67%), employed (*n* = 96, 65.31%), single (*n* = 83, 56.46%) and had some/completed post-secondary education (*n* = 118, 80.27%). The mean age was 30.81 years (*SD* = 8.94, see Table 1).

**Table 1:** Descriptive statistics.

Variable	*M*	*SD*	Min	Max	%	*n*
Age (in years)	30.81	8.94	19.00	57.00		147
Gender						
Men					70.07	103
Women					27.21	40
Non-binary/Other*					2.72	4
Ethnicity						
White/European					66.67	98
Non-white/Non European					33.33	49
Employment status						
Student					19.73	29
Employed					65.31	96
Other					14.97	22
Income						
Less than $24,999 - $49,999					50.34	74
$50,000 - $99,999					36.73	54
Greater than $100,000					12.93	19
Education						
Grade School					10.88	16
Some/Completed Post-Secondary					80.27	118
Post-graduate					8.84	13
Marital Status/Common-Law						
Yes					43.54	64
No					56.46	83

*N* = 147. Some totals do not sum to *N* = 147 due to missing values.

*Excluded from analyses due to the small size of the subsample.

### Substance use

Participants had used most legal (i.e., alcohol, tobacco, cannabis) substances (*M* = 2.55, *SD* = .70). Although the average illegal (i.e., sedatives, opioids, cocaine, amphetamines, hallucinogens, inhalants/solvents) substance use score was *M* = 1.56 substances (*SD* = 1.72), 39.46% of the sample had used none of these substances in their lifetime. On average, participants had used approximately a total of 4 substances (*M* = 4.12, *SD* = 2.00) over their lifetime. Regarding cannabis use, the most frequently used primary method was joints (*n* = 41, 27.89%). Among participants that currently used cannabis, most participants indicated that they used cannabis daily (*n* = 30, 20.41%), yearly (*n* = 34, 23.13%) or weekly (*n* = 35, 23.81%). Participants were approximately 19 years of age when they first tried cannabis (*M* = 19.49 years, *SD* = 5.96), had used cannabis approximately 9 days of the past month (*M* = 8.90, *SD* = 11.08), and had used cannabis for approximately 8 years in total (*M* = 7.87, *SD* = 7.22). DSM-5 CUD scores reflected, on average, symptoms of a mild CUD (*M* = 3.63, *SD* = 3.30).

Q-LES-Q-SF percent maximum scores showed that the mean percent maximum score for quality of life was 58.35% (*M =* .58, *SD =* .19). Control or community samples tend to have percent maximum scores between 70.00% and 100.00% [36, 37]. Thus, the mean Q-LES-Q-SF percent maximum score in the current study is lower, as is typically reported in clinical populations [37]. DSM-5 CC scores indicated moderate externalizing (*M =* 1.98, *SD =* 1.49) and internalizing (*M =* 3.05, *SD =* 2.34) symptoms, but lower levels of mania and psychosis symptoms (*M =* 1.69, *SD =* 1.55; see Appendix 1 in S1 File).

### Latent profile analysis

Parameter estimates to select the number of profiles was determined by examining various indices such as the Bootstrap Likelihood Ratio Test (BLRT), Akaike Information Criteria (AIC), and Bayesian Information Criteria (BIC), as well as entropy values, which indicate profile separation. In a review of LPA literature, Spurk et al. [38] indicated that BIC, BLRT, and entropy values should be prioritized to assess model fit, with higher entropy values, significant BLRT *p*-values, and lower BIC and AIC values indicating superior fit. All profiles had a significant BLRT statistic. BIC and AIC values pointed toward a four-model solution and entropy values were only minimally superior in the two-profile model compared to the four-profile model. Based on these parameters, a four-model solution was chosen (see Appendix 2 in S1 File).

#### Profile characteristics

Profile means for study measures were examined to facilitate defining profile characteristics (see Table 2). A Chi-Square Test for Independence with Bonferroni corrected *p* values (*p* < .006) was conducted to examine profile differences on categorical measures (see Appendix 3 in S1 File). A Bonferroni corrected (*p* < .005) one-way between groups ANOVA examining group differences in age, externalizing symptoms, internalizing symptoms, mania and psychosis symptoms, quality of life, past week DSM CUD, polysubstance use, average monthly cannabis use (in days), age when participants first tried cannabis, and years participants had used cannabis was also conducted (see Appendix 4 in S1 File).

**Table 2:** Descriptive statistics for profile defining & dependent variables.

Variables	Rapidly Escalating High Risk (*n* = 40)	Low Risk (*n* = 62)	Long-Term High Severity (*n* = 35)	Long-Term Lower Severity (*n* = 10)
	*M (SD)*	*M (SD)*	*M (SD)*	*M (SD)*
Age	28.03(7.10)	31.82(8.56)	27.09 (5.07)	48.70 (5.77)
Substance Use				
DSM-5 CUD	5.96(2.70)	1.63(2.27)	4.70(3.31)	1.13(2.10)
Poly Use	4.58(1.97)	3.41(1.77)	4.77(2.07)	4.40(2.01)
Frequency (days in past month)	4.85(4.80)	2.40(4.38)	26.83(4.73)	2.60(4.58)
Age of first use	19.86(5.23)	20.73(6.94)	16.71(3.18)	20.20(7.02)
Total years	4.69(3.61)	6.02(3.97)	8.89(5.13)	28.20(6.89)
Mental Health				
External	3.53(.85)	1.02(1.03)	2.09(1.34)	1.40(1.26)
Internal	5.28(.82)	1.31(1.50)	3.80(2.15)	2.40(2.76)
Mania & Psychosis	3.50(.82)	.81(1.01)	1.46(1.38)	.80(1.03)
Quality of Life	.57(.19)	.66(.17)	.49(.17)	.50(.23)
Decrease Attempts	12.10(10.02)	4.23(4.92)	5.42(4.68)	3.44(3.71)
Quit Attempts	3.43(3.44)	1.73(2.20)	1.59(2.47)	2.67(3.32)

In summary, profile 1 was comprised of more individuals who used cannabis weekly (30.00%). Furthermore, individuals in profile 1 had significantly higher externalizing (*M* = 3.53, *SD =* .85), internalizing (*M* = 5.28, *SD =* .82), and mania and psychosis (*M* = 3.50, *SD =* .82) symptoms compared to other profiles. Profile 1 also had the highest mean DSM-5 CUD scores (*M =* 5.96, *SD =* 2.70), and had used cannabis for the least number of years (*M =* 4.69, *SD =* 3.61). More individuals in profile 1 had started using cannabis after legalization (56.41%). Based on these characteristics, profile 1 was labelled the rapidly escalating high-risk group.

Profile 2 was characterized by more individuals who were not currently using cannabis (30.65%). Individuals in profile 2 had the least amount of lifetime polysubstance use (*M =* 3.41, *SD =* 1.77), the highest mean age of first use (*M =* 20.73, *SD =* 6.94), and exhibited the lowest scores on externalizing (*M =* 1.02, *SD =* 1.03) and internalizing mental health measures (*M =* 1.31, *SD =* 1.50). Not surprisingly, on average, participants in this profile reported 1.63 (*SD* = 2.27) CUD symptoms. More individuals in profile 2 had started using cannabis before legalization (56.45%). Profile 2 also had the highest mean quality of life scores (*M =* .66, *SD =* .17). Based on these results, profile 2 was labelled the low-risk group.

Profile 3 was characterized by more individuals who used cannabis on a daily basis (77.14%). Individuals in profile 3 had the lowest mean age (*M =* 27.09, *SD =* 5.07), highest amount of polysubstance use (*M =* 4.77, *SD =* 2.07), and had used cannabis for significantly more days on average in the past month compared to other profiles (*M =* 26.83, *SD =* 4.73). Profile 3 also included individuals who had the lowest mean age of first use of cannabis (*M =* 16.71, *SD =* 3.18), and had the lowest mean quality of life (*M =* .49, *SD =* .17). Most individuals in profile 3 had started using cannabis prior to legalization (80.00%). Based on these results, profile 3 was labelled the long-term high severity profile.

Profile 4 included more individuals who were currently using cannabis on a yearly (50.00%) or monthly basis (20.00%). Participants in profile 4 had a significantly higher mean age compared to other profiles (*M =* 48.70, *SD =* 5.77). Individuals in profile 4 had used cannabis for the most years on average (*M =* 28.20, *SD =* 6.89), and had the lowest mean mania and psychosis mental health scores (*M =* .80, *SD =* 1.03). All participants in profile 4 started using cannabis prior to legalization (100.00%). On average, participants in this profile reported 1.13 (*SD* = 2.10) CUD symptoms Based on these findings, profile 4 was labelled the long-term lower severity group.

### Profiles and cannabis cessation

A Bonferonni corrected (*p* < .025) ANCOVA (controlling for participants’ age) was conducted to examine profile differences on the number of times individuals had attempted to decrease or quit cannabis. Bonferroni’s post hoc tests were also reported (see Appendix 5 in S1 File). To examine the trajectory of cannabis use after the last attempt to decrease or quit cannabis, a Chi-Square Test of Independence was conducted to see how participants’ use had changed and whether this differed by profile. Profiles were also compared on whether they sought formal treatment (i.e., individual/online counselling or Contingency Management [CM] programs) or non-formal (e.g., support groups, books, visual media, replacing cannabis with food, drug, or activity) support for their cannabis use (see Table 3).

**Table 3:** Results of chi-square test of independence for four LPA profiles–Trajectory of cannabis use & treatment.

Variable	Rapidly Escalating High Risk		Low Risk		Long Term High Severity		Long Term Lower Severity			χ2
	*n*	%	*n*	%	*n*	%	*n*		*%*	
After your last attempt to decrease or quit cannabis use, did your use:										34.45**
Completely stop	8	13.79	39	67.24	8	13.79	3		5.17	
Decreased but did not completely stop	22	33.85	21	32.31	15	23.08	7		10.77	
Stayed the same	5	35.71	2	14.29	7	50.00	0		0.00	
Got worse	2	33.33	0	0.00	4	66.67	0		0.00	
Treatment										39.19**
Formal Treatment	31	77.50	23	37.13	10	29.41	2		20.00	
Informal Treatment/Not used	9	22.50	39	62.90	24	70.59	8	80.00		

** *p* < .001.

Participants in the rapidly escalating high risk profile had attempted to decrease their cannabis use more times compared to other profiles (*M =* 12.10, *SD =* 10.02), more individuals in this profile indicated that their use had decreased but had not completely stopped after their last attempt (33.85%), and had accessed formal treatment for their cannabis use (77.50%).

Most participants in the low-risk group indicated that their use had completely stopped after their last cessation attempt (67.24%), and similar to the rapidly escalating profile, 32.31% of individuals in the low risk profile indicated their use had decreased but did not completely stop after their last cessation attempt.

More participants in the long-term high severity profile indicated that their cannabis use had stayed the same (50.00%) or gotten worse (66.67%) after their last cessation attempt. Participants in this profile were also more likely to have not accessed formal treatment (70.59%) and had the lowest mean number of quit attempts (*M =* 1.59, *SD =* 2.47).

Individuals in the long-term lower severity profile were more likely to say that their use had decreased but did not completely stop after their last cessation attempt (10.77%). Additionally, most individuals in this profile had not accessed formal treatment (80.00%) and had the lowest mean number of decrease attempts (*M =* 3.44, *SD =* 3.71).

## Discussion

Most of the research on cannabis users in Canada has been conducted prior to legalization of cannabis in 2018 [18, 39]. Furthermore, prior research has demonstrated that individuals with CUD experience difficulties achieving cessation [40] but that there is a lack of research examining how similar groups of cannabis users attempt to reduce or abstain from cannabis. Thus, the current study aimed to address gaps in the existing research by examining individual differences in patterns of cannabis use and how these may be related to cannabis cessation. For this purpose, emerging cannabis profiles were compared on the number of times they had attempted to decrease and quit cannabis and whether they had accessed formal treatment to assist them.

Overall, mostly as hypothesized, profiles fell into high-severity and lower-severity categories based on length of cannabis use, frequency of use and DSM-5 CUD symptoms. However, two higher severity profiles emerged: rapidly escalating high-risk and long-term high-severity profiles as well as two lower-severity profiles: low-risk and long-term lower-severity. The rapidly escalating high-risk profile suggested rapidly worsening cannabis use. Specifically, individuals in the rapidly escalating profile had used on average for less than 5 years but reported CUD symptoms in the severe range. Participants in the rapidly escalating high-risk group also had the most severe internalizing, externalizing and mania and psychosis symptoms. Lastly, these individuals also had a higher level of drug experimentation, averaging use of 5 substances in their lifetime, although a large proportion (56%) had started using cannabis after legalization.

In contrast, the long-term high severity group had used cannabis for longer than the rapidly escalating group, approximately 9 years (80% had started using before legalization), and used cannabis more frequently (77.14% used daily). Furthermore, this group reported less mental health symptoms but also lower quality of life than the rapidly escalating group. However, the two high risk groups were not different on polysubstance use, and CUD symptoms, with both groups having higher lifetime polysubstance use scores and moderate/high CUD severity. Despite similarities between these two severe groups of cannabis users, these profiles were significantly different in their reported attempts at reduction or abstinence of cannabis. In comparison to the long-term severity profile, the rapidly escalating profile reported double the number of abstinence attempts (1.59 versus 3.43 attempts, respectively) and of reduction attempts (5.42 versus 12.10 attempts). Furthermore, the rapidly escalating profile was more likely to report accessing formal counselling or contingency management services (77%) in comparison to the long-term severity profile (16%).

The findings of the current study suggest that although both high severity profiles may have higher intensity treatment needs given their symptoms of CUD, the long-term severity group is not attempting cannabis reduction and abstinence as often and is not accessing formal support to assist with their recovery. Furthermore, when asked about their latest recovery attempt, participants in the long-term high severity sample were more likely to say that their use had gotten worse. This is concerning given that this group had quality of life scores averaging 49% out of a maximum of 100%. Although it is unclear why these two profiles of cannabis users are different in their reduction and cessation attempts, the current findings suggests that more treatment initiatives are needed to target long-term daily cannabis users who started using during their teenage years. Further research should investigate whether specific factors such as motivations for use may contribute to the finding that long-term high severity users were less likely to attempt cannabis reduction or abstinence. Specifically, it has been found that in current cannabis users reporting at least one cannabis cessation attempt, those with difficulties tolerating distress were more likely to use cannabis for coping motives and reported more barriers to cannabis cessation [41]. It is possible that rapidly escalating high risk and long-term higher severity groups are using cannabis for different reasons and that these motives for use affect motivation for cannabis reduction/cessation as well as successful outcomes. Furthermore, polysubstance use may be impeding cannabis recovery. It is common for individuals to use cannabis to modulate the effects (i.e., withdrawal or ‘coming down’) of another drug, such as stimulants or opioids [42]. However, mixed findings have been reported with regard to cannabis users reporting increases or reduction of use of other substances during cannabis cessation [13, 43], suggesting the need for further research.

In contrast, individuals in the low risk profile exhibited between 1 to 2 CUD symptoms, used on average 2 days per week (although this profile include participants who no longer used cannabis) and reported the lowest mental health symptoms overall. Although they rarely (21%) accessed formal substance use services, they were the most likely to report that their use completely stopped after their latest attempt. However, as Hughes and colleagues [13] found, cannabis cessation attempts often do not last more than a few days. Therefore, although most individuals in this low risk group may not require formal support, some may benefit from harm reduction initiatives, such as the Canadian Lower Risk Cannabis Use Guidelines, which outline several recommendations for cannabis users to make informed decisions about cannabis use and reduce the risks of severe harm [44]. Finally, the remaining profile, the long-term lower severity group consisted of a middle-aged group of cannabis users, who had used cannabis for approximately 28 years. Although they experienced few DSM-5 CUD symptoms, they were reporting lower quality life like the long-term high severity users, which is concerning. Although quality of life can decrease with age [45], further research is warranted to investigate why long-term cannabis users are reporting lower satisfaction with life. Given that some cannabis users report using cannabis for medicinal purposes, some individuals in this profile may be experiencing acute or chronic medical conditions [46].

Strengths of the current study include an analysis of cannabis use patterns and their association with cannabis reduction and cessation attempts in a small but diverse sample of cannabis users. Specifically, we recruited participants with various patterns of cannabis use and with demographic characteristics that are representative of Canadian cannabis users [47]. The current study also has several limitations. The sample size was limited by the recruitment of cannabis users who reported at least one attempt at cannabis cessation. Furthermore, the use of a cross-sectional design does not allow for causal inferences and self-reported data may have been affected by memory recall bias. A cross-lagged longitudinal design would allow for the examination of the causal relationships between patterns of cannabis use and cannabis cessation. Furthermore, future research could investigate why cannabis users in the different profiles decide to reduce or abstain cannabis (i.e., differences in attitudes and motivations) and whether treatment matching to distinct cannabis interventions improves outcomes.

## Supporting information

S1 File Descriptive statistics, latent profile analysis, chi-square tests, and post-hoc multiple comparisons. (DOCX)
