# Supplementary material for: Profiles of cannabis users and impact on cannabis cessation
Source: PLoS One. 2024 Jun 11;19(6):e0305088. doi: 10.1371/journal.pone.0305088 (PMC11166302; doi:10.1371/journal.pone.0305088)
Supplement: S1 File — (DOCX) [file pone.0305088.s001.docx]

**Appendix 1. Descriptive Statistics for Study Measures in Overall Sample**

| Variable | *M* | *SD* | Min | Max | % | *n* |
| --- | --- | --- | --- | --- | --- | --- |
| Legal Substance Use Score | 2.55 | .70 | 0 | 3 |  | 146 |
| Illegal/Non-Prescribed Substance Use Score | 1.56 | 1.72 | 0 | 6 |  | 147 |
| Polysubstance Use | 4.12 | 2.00 | 0 | 9 |  | 146 |
| Cannabis Use Frequency (DFAQ-CU)^*^ |  |  |  |  |  |  |
| Daily |  |  |  |  | 20.41 | 30 |
| Weekly |  |  |  |  | 23.81 | 35 |
| Monthly |  |  |  |  | 7.48 | 11 |
| Yearly |  |  |  |  | 23.13 | 34 |
| Less than once a year |  |  |  |  | 8.84 | 13 |
| DFAQ-CU |  |  |  |  |  |  |
| Age of first use | 19.49 | 5.96 | 7.00 | 45.00 |  | 144 |
| Frequency (days in past month) | 8.90 | 11.08 | .00 | 31.00 |  | 147 |
| Total years | 7.87 | 7.22 | 1.00 | 40.00 |  | 146 |
| DSM-5 CUD |  |  |  |  |  |  |
| Past Week DSM symptoms | 3.63 | 3.30 | .00 | 11.00 |  | 87 |
| Q-LES-Q-SF |  |  |  |  |  |  |
| Percent Maximum Score | .58 | .19 | .10 | 1.00 |  | 145 |
| DSM-5 CC Measure |  |  |  |  |  |  |
| Internalizing | 3.05 | 2.34 | .00 | 6.00 |  | 147 |
| Externalizing | 1.98 | 1.49 | .00 | 4.00 |  | 147 |
| Mania & Psychosis | 1.69 | 1.55 | .00 | 4.00 |  | 147 |

Some totals do not sum to N = 147 due to missing values. DFAQ-CU = Daily Sessions, Frequency, Age of Onset, and Quantity of Cannabis Use. DSM-5 CC Measure = DSM-5 Self-Rated Level 1 Cross-Cutting Symptom Measure-Adult. DSM-5 CUD = Diagnostic and Statistical Manual of Mental Disorders, Fifth Edition Cannabis Use Disorder. Q-LES-Q-SF = Quality of Life Enjoyment and Satisfaction Questionnaire.

^*^Totals do not sum to N = 147 as this only includes participants who currently use cannabis.

**Appendix 2. Latent Profile Analysis Summary**

| Profile | 2 | 3 | 4 |
| --- | --- | --- | --- |
| Maximum Loglikelihood | -3427.66 | -3376.11 | -3320.93 |
| AIC | 6917.33 | 6836.22 | 6747.85 |
| BIC | 7010.03 | 6961.81 | 6906.35 |
| Entropy | 0.97 | 0.94 | 0.96 |
| Bootstrap Likelihood Ratio Test (BLRT) | 337.44 | 103.11 | 110.36 |
| Bootstrap Likelihood Ratio Test p Value | p = .01 | p = .01 | p = .01 |
| Breakdown of Participants per Profile (n) | 77/70 | 62/74/11 | 40/62/35/10 |

1. AIC = Akaike Information Criterion; BIC = Bayesian Information Criterion

**Appendix 3. Results of Chi-Square Test of Independence for Four LPA Profiles – Demographics & Cannabis Use**

| Variable | Profile 1 Rapidly Escalating High Risk |  | Profile 2 Low Risk |  | Profile 3 Long-Term High Severity |  | Profile 4 Long-Term low Severity |  | χ2 |
| --- | --- | --- | --- | --- | --- | --- | --- | --- | --- |
|  | *n* | % | *n* | % | *n* | % | *n* | *%* |  |
| Ethnicity |  |  |  |  |  |  |  |  | 3.22 |
| Caucasian | 28 | 28.57 | 37 | 37.76 | 27 | 27.55 | 6 | 6.12 |  |
| Other | 12 | 24.49 | 25 | 51.02 | 8 | 16.33 | 4 | 8.16 |  |
| Gender |  |  |  |  |  |  |  |  | 6.66 |
| Female | 8 | 20.00 | 19 | 47.50 | 13 | 32.50 | 0 | 0.00 |  |
| Male | 31 | 30.10 | 42 | 40.78 | 21 | 20.39 | 9 | 8.74 |  |
| Occupation |  |  |  |  |  |  |  |  | 12.22 |
| Student | 13 | 44.83 | 9 | 31.03 | 7 | 24.14 | 0 | 0.00 |  |
| Employed | 23 | 23.96 | 45 | 46.88 | 22 | 22.92 | 6 | 6.25 |  |
| Other | 4 | 18.18 | 8 | 36.36 | 6 | 27.27 | 4 | 18.18 |  |
| Education |  |  |  |  |  |  |  |  | 7.49 |
| Grade | 2 | 12.50 | 7 | 43.75 | 7 | 43.75 | 0 | 0.00 |  |
| Post Secondary | 34 | 28.81 | 50 | 42.37 | 24 | 20.34 | 10 | 8.47 |  |
| Postgraduate | 4 | 30.77 | 5 | 38.46 | 4 | 30.77 | 0 | 0.00 |  |
| Income |  |  |  |  |  |  |  |  | 8.98 |
| Less than $25,000 – 49,999 | 20 | 27.03 | 24 | 32.43 | 24 | 32.43 | 6 | 8.11 |  |
| $50,000 – 99,999 | 16 | 29.63 | 27 | 50.00 | 8 | 14.81 | 3 | 5.56 |  |
| >$100,000 | 4 | 21.05 | 11 | 57.89 | 3 | 15.79 | 1 | 5.26 |  |
| Relationship |  |  |  |  |  |  |  |  | 3.08 |
| Yes | 20 | 31.25 | 29 | 45.31 | 11 | 17.19 | 4 | 6.25 |  |
| No | 20 | 24.10 | 33 | 39.76 | 24 | 28.92 | 6 | 7.23 |  |
| Average Frequency |  |  |  |  |  |  |  |  | 121.46** |
| I do not use cannabis | 4 | 10.00 | 19 | 30.65 | 1 | 2.86 | 0 | 0.00 |  |
| Less than once a year | 2 | 5.00 | 10 | 16.13 | 0 | 0.00 | 1 | 10.00 |  |
| Yearly | 14 | 35.00 | 15 | 24.19 | 0 | 0.00 | 5 | 50.00 |  |
| Monthly | 7 | 17.50 | 2 | 3.23 | 0 | 0.00 | 2 | 20.00 |  |
| Weekly | 12 | 30.00 | 15 | 24.19 | 7 | 20.00 | 1 | 10.00 |  |
| Daily | 1 | 2.50 | 1 | 1.61 | 27 | 77.14 | 1 | 10.00 |  |
| Legalization |  |  |  |  |  |  |  |  | 17.29** |
| Post Legalization | 22 | 56.41 | 27 | 43.45 | 7 | 20.00 | 0 | 0.00 |  |
| Pre/During Legalization | 17 | 43.59 | 35 | 56.45 | 28 | 80.00 | 10 | 100.00 |  |

^**^sig at *p* < .001

**Appendix 4. Bonferroni Post-Hoc Multiple Comparisons for Profile Defining Variables**

|  |  |  |  |  | 95% Confidence Interval | |
| --- | --- | --- | --- | --- | --- | --- |
| Variable | (I) Latent Class Cluster | (J) Latent Class Cluster | Mean Difference (I-J) | Std. Error | Lower Bound | Upper Bound |
| Age | 1 | 2 | -3.80 | 1.48 | -7.76 | .16 |
|  |  | 3 | .94 | 1.69 | -3.58 | 5.46 |
|  |  | 4 | -20.68** | 2.58 | -27.58 | -13.77 |
|  | 2 | 3 | 4.74 | 1.54 | .61 | 8.86 |
|  |  | 4 | -16.88** | 2.49 | -23.53 | -10.23 |
|  | 3 | 4 | -21.61** | 2.62 | -28.61 | -14.61 |
| DSM-5 CC Externalizing | 1 | 2 | 2.51** | .22 | 1.92 | 3.10 |
|  |  | 3 | 1.44** | .25 | .77 | 2.11 |
|  |  | 4 | 2.13** | .38 | 1.10 | 3.15 |
|  | 2 | 3 | -1.07** | .23 | -1.68 | -.46 |
|  |  | 4 | -.38 | .37 | -1.37 | .60 |
|  | 3 | 4 | .69 | .39 | -.35 | 1.73 |
| DSM-5 CC Internalizing | 1 | 2 | 3.97** | .33 | 3.07 | 4.86 |
|  |  | 3 | 1.48* | .38 | .45 | 2.50 |
|  |  | 4 | 2.88** | .58 | 1.31 | 4.44 |
|  | 2 | 3 | -2.49** | .35 | -3.43 | -1.56 |
|  |  | 4 | -1.09 | .56 | -2.60 | .41 |
|  | 3 | 4 | 1.40 | .59 | -.18 | 2.98 |
| DSM-5 CC Mania & Psychosis | 1 | 2 | 2.69** | .22 | 2.12 | 3.27 |
|  |  | 3 | 2.04** | .25 | 1.38 | 2.70 |
|  |  | 4 | 2.70** | .38 | 1.69 | 3.71 |
|  | 2 | 3 | -.65 | .23 | -1.25 | -.05 |
|  |  | 4 | .01 | .36 | -.96 | .98 |
|  | 3 | 4 | .66 | .38 | -.36 | 1.68 |
| Q-LES-Q-SF | 1 | 2 | -.08 | .04 | -.18 | .01 |
|  |  | 3 | .08 | .04 | -.03 | .19 |
|  |  | 4 | .07 | .06 | -.10 | .24 |
|  | 2 | 3 | .17** | .04 | .07 | .27 |
|  |  | 4 | .15 | .06 | -.01 | .31 |
|  | 3 | 4 | -.01 | .06 | -.19 | .16 |

DFAQ-CU = Daily Sessions, Frequency, Age of Onset, and Quantity of Cannabis Use. DSM-5 CC Measure = DSM-5 Self-Rated Level 1 Cross-Cutting Symptom Measure-Adult. DSM-5 CUD = Diagnostic and Statistical Manual of Mental Disorders, Fifth Edition Cannabis Use Disorder. Q-LES-Q-SF = Quality of Life Enjoyment and Satisfaction Questionnaire.

**p* <.005

** *p* <.001

**Appendix 4. Bonferroni Post-Hoc Multiple Comparisons for Profile Defining Variables (Cont.)**

|  |  |  |  |  | 95% Confidence Interval | |
| --- | --- | --- | --- | --- | --- | --- |
| Variable | (I) Latent Class Cluster | (J) Latent Class Cluster | Mean Difference (I-J) | Std. Error | Lower Bound | Upper Bound |
| DSM-5 CUD | 1 | 2 | 4.34** | .70 | 2.46 | 6.22 |
|  |  | 3 | 1.26 | .78 | -.86 | 3.38 |
|  |  | 4 | 4.84** | 1.07 | 1.94 | 7.73 |
|  | 2 | 3 | -3.08** | .76 | -5.12 | -1.03 |
|  |  | 4 | .50 | 1.05 | -2.34 | 3.34 |
|  | 3 | 4 | 3.58 | 1.11 | .57 | 6.58 |
| Polysubstance Use | 1 | 2 | 1.17 | .39 | .12 | 2.21 |
|  |  | 3 | -.20 | .44 | -1.39 | .99 |
|  |  | 4 | .18 | .68 | -1.64 | 1.99 |
|  | 2 | 3 | -1.36 | .41 | -2.45 | -.27 |
|  |  | 4 | -.99 | .65 | -2.74 | .76 |
|  | 3 | 4 | .37 | .69 | -1.47 | 2.21 |
| Frequency (days in past month) | 1 | 2 | 2.45 | .93 | -.04 | 4.94 |
|  |  | 3 | -21.98** | 1.06 | -24.82 | -19.14 |
|  |  | 4 | 2.25 | 1.62 | -2.09 | 6.59 |
|  | 2 | 3 | -24.43** | .97 | -27.02 | -21.83 |
|  |  | 4 | -.20 | 1.56 | -4.38 | 3.99 |
|  | 3 | 4 | 24.23** | 1.65 | 19.82 | 28.63 |
| Age of first use | 1 | 2 | -.86 | 1.20 | -4.08 | 2.36 |
|  |  | 3 | 3.15 | 1.37 | -.51 | 6.81 |
|  |  | 4 | -.34 | 2.07 | -5.87 | 5.20 |
|  | 2 | 3 | 4.01 | 1.23 | .73 | 7.29 |
|  |  | 4 | .53 | 1.98 | -4.76 | 5.81 |
|  | 3 | 4 | -3.49 | 2.08 | -9.05 | 2.08 |
| Total years | 1 | 2 | -1.32 | .90 | -3.74 | 1.09 |
|  |  | 3 | -4.19** | 1.03 | -6.95 | -1.44 |
|  |  | 4 | -23.51** | 1.57 | -27.70 | -19.31 |
|  | 2 | 3 | -2.87 | .93 | -5.37 | -.37 |
|  |  | 4 | -22.18** | 1.51 | -26.21 | -18.15 |
|  | 3 | 4 | -19.31** | 1.59 | -23.56 | -15.07 |

DFAQ-CU = Daily Sessions, Frequency, Age of Onset, and Quantity of Cannabis Use. DSM-5 CC Measure = DSM-5 Self-Rated Level 1 Cross-Cutting Symptom Measure-Adult. DSM-5 CUD = Diagnostic and Statistical Manual of Mental Disorders, Fifth Edition Cannabis Use Disorder. Q-LES-Q-SF = Quality of Life Enjoyment and Satisfaction Questionnaire.

** *p* < .001

**Appendix 5. Bonferroni Post-Hoc Pairwise Comparisons for Times Decreased Cannabis.**

|  |  |  |  |  | 95% Confidence Interval | |
| --- | --- | --- | --- | --- | --- | --- |
| Variable | (I) Latent Class Cluster | (J) Latent Class Cluster | Mean Difference (I-J) | Std. Error | Lower Bound | Upper Bound |
| How many times have you attempted to decrease your cannabis use? | 1 | 2 | 7.96** | 1.47 | 4.01 | 11.90 |
|  |  | 3 | 6.63* | 1.74 | 1.97 | 11.29 |
|  |  | 4 | 9.20* | 2.86 | 1.52 | 16.88 |
|  | 2 | 3 | -1.33 | 1.55 | -5.49 | 2.84 |
|  |  | 4 | 1.24 | 2.61 | -5.76 | 8.23 |
|  | 3 | 4 | 2.56 | 2.96 | -5.38 | 10.50 |

**p* < .025

***p* < .001
